# Unraveling the role of VLDL in the relationship between type 2 diabetes and coronary atherosclerosis: a Mendelian randomization analysis

**DOI:** 10.3389/fcvm.2023.1234271

**Published:** 2023-10-30

**Authors:** Wenshuai Feng, Liuli Guo, Yiman Liu, Ming Ren

**Affiliations:** ^1^ College of Traditional Chinese Medicine, Tianjin University of Traditional Chinese Medicine, Tianjin, China; ^2^ Baokang Hospital Affiliated to Tianjin University of Traditional Chinese Medicine, Tianjin, China

**Keywords:** type 2 diabetes, very low-density lipoprotein, coronary atherosclerosis, Mendelian randomization, mediation pathway

## Abstract

**Background:**

The causal link between Type 2 diabetes (T2D) and coronary atherosclerosis has been established through wet lab experiments; however, its analysis with Genome-wide association studies (GWAS) data remains unexplored. This study aims to validate this relationship using Mendelian randomization analysis and explore the potential mediation of VLDL in this mechanism.

**Methods:**

Employing Mendelian randomization analysis, we investigated the causal connection between T2D and coronary atherosclerosis. We utilized GWAS summary statistics from European ancestry cohorts, comprising 23,363 coronary atherosclerosis patients and 195,429 controls, along with 32,469 T2D patients and 183,185 controls. VLDL levels, linked to SNPs, were considered as a potential mediating causal factor that might contribute to coronary atherosclerosis in the presence of T2D. We employed the inverse variance weighted (IVW), Egger regression (MR-Egger), weighted median, and weighted model methods for causal effect estimation. A leave-one-out sensitivity analysis was conducted to ensure robustness.

**Results:**

Our Mendelian randomization analysis demonstrated a genetic association between T2D and an increased coronary atherosclerosis risk, with the IVW estimate at 1.13 [95% confidence interval (CI): 1.07–1.20]. Additionally, we observed a suggestive causal link between T2D and VLDL levels, as evidenced by the IVW estimate of 1.02 (95% CI: 0.98–1.07). Further supporting lipid involvement in coronary atherosclerosis pathogenesis, the IVW-Egger estimate was 1.30 (95% CI: 1.06–1.58).

**Conclusion:**

In conclusion, this study highlights the autonomous contributions of T2D and VLDL levels to coronary atherosclerosis development. T2D is linked to a 13.35% elevated risk of coronary atherosclerosis, and within T2D patients, VLDL concentration rises by 2.49%. Notably, each standard deviation increase in VLDL raises the likelihood of heart disease by 29.6%. This underscores the significant role of lipid regulation, particularly VLDL, as a mediating pathway in coronary atherosclerosis progression.

## 1. Introduction

### 1.1. Unraveling the pathogenesis of type 2 diabetes and coronary atherosclerosis

Type 2 diabetes (T2D) stands as a pervasive metabolic disorder affecting a substantial global population (1). Its intricate etiology, devoid of a definitive cure, compels a focus on symptom alleviation and complication prevention (2). Unfortunately, the prevalent chronic complications of T2D, primarily impacting cardiovascular and nerves, pose significant morbidity and mortality risks (3). Among these complications, coronary atherosclerosis emerges as a formidable adversary—characterized by plaque accumulation in the coronary arteries nourishing the heart. Given the pronounced atherogenic tendencies of T2D patients, exploring the mechanisms behind the T2D-coronary atherosclerosis nexus becomes pivotal (4). Thus, deciphering the pathophysiological intricacies driving coronary atherosclerosis in T2D patients is imperative, enabling the formulation of efficacious prevention and management strategies.

In essence, the pervasive prevalence of T2D and its consequential coronary atherosclerosis mandate an in-depth inquiry into the underlying mechanisms. Enhancing our comprehension of T2D's pathophysiology and its cascading complications promises more potent approaches to prevent, manage, and enhance patient well-being.

### 1.2. Mendelian randomization

**Figure 1:**
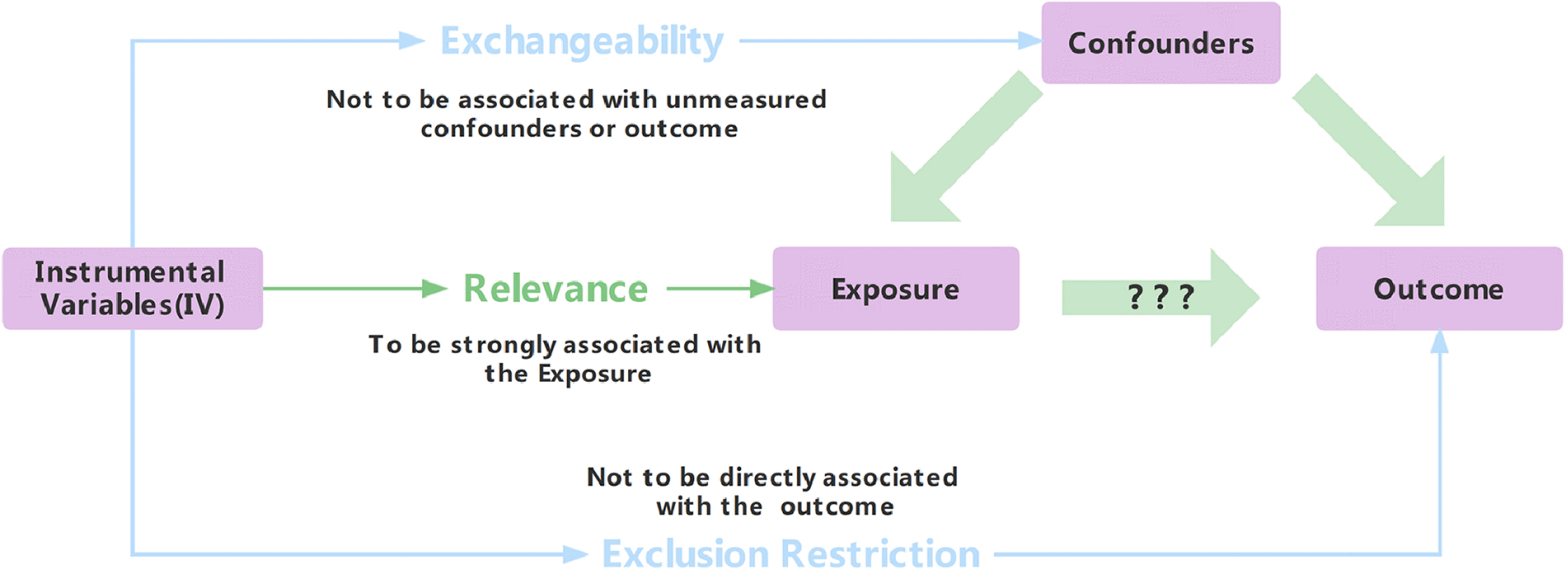
Three assumptions about instrumental variables (IV).

Traditional statistical methods for exploring cause-and-effect relationships are flawed due to bias and confounding (5). Mendelian randomization (MR) mitigates confounding and reverse causality issues. Instrumental variables (IVs), linked to exposures but not outcomes or confounding, underpin MR (6–8). Three assumptions—relevance, exchangeability, and exclusion restriction—support MR validity (Figure 1) (9). Despite benefits, strict IV requirements limit MR's use. Genome-wide association studies introduce single-nucleotide polymorphisms (SNPs) as robust IVs (10). SNPs serve as popular IVs (11) and uncover novel genetic determinants.

Despite challenges like sample size demands and pleiotropy, MR promises to refine our grasp of complex diseases and their influences. Leveraging MR strengthens causal conclusions, enhancing intervention and management strategies.

### 1.3. The link between T2D and coronary atherosclerosis

T2D is a pervasive metabolic disorder with a significant global impact. While established links exist between T2D and coronary atherosclerosis (12) gaps remain in comprehending the intricate mechanisms that intricately connect the two (13). The conventional focus on risk factors like hypertension, obesity, and dyslipidemia partially explains the relationship, but an evolving body of research suggests the direct involvement of T2D in atherogenesis, notably impacting coronary atherosclerosis's pathogenesis (14). Central to this relationship is chronic hyperglycemia, a hallmark of T2D, which amplifies cardiovascular risks (15). It impairs endothelium-dependent vasodilation, compromising vascular health (16). Intriguingly, the accumulation of advanced glycation end products (AGEs) amid hyperglycemia plays a pivotal role in T2D-driven coronary atherosclerosis development (17). AGEs activate receptors, inciting inflammation, and cell proliferation, further exacerbating atherosclerosis (18).

### 1.4. VLDL's role in T2D-coronary atherosclerosis

#### 1.4.1. VLDL is linked to T2D

Elevated fatty acid levels due to hyperinsulinemia are well-documented contributors to metabolic disorders, including T2D (19). These fatty acids trigger immune responses, inducing inflammatory cytokines like TNF-α, IL-1, and IL-6 (20). This inflammatory milieu drives insulin resistance, disrupts glucose homeostasis, and fosters T2D. Moreover, inhibiting the liver X receptor escalates cholesterol accumulation, inducing CRP, plasminogen inhibitor-1, serum amyloid, fostering fibrinogen synthesis, and hypercoagulability (21). These cytokines catalyze VLDL and free fatty acid production, exacerbating lipid disorders, promoting arterial lipid deposition, and augmenting atherosclerotic risk (22). The complexity of these interactions underscores the multifaceted nature of metabolic disorder pathogenesis, necessitating a comprehensive understanding.

#### 1.4.2. VLDL's role in causing coronary atherosclerosis

Notably, dyslipidemia, particularly the presence of very low-density lipoprotein (VLDL) and elevated triglyceride (TG) levels, has been linked to coronary atherosclerosis (23). It is revealed that elevated levels of very-low-density lipoprotein cholesterol (VLDL-C) are associated with an increased risk of major adverse limb events (MALE) in patients with cardiovascular disease (24). However, there is no correlation between VLDL-C levels and major adverse cardiovascular events (MACE) or all-cause mortality, even after accounting for established risk factors such as LDL-C and lipid-lowering medication (24). Postprandial remnant lipoproteins, especially VLDL remnants, play a significant role in the initiation and progression of atherosclerosis (25). The increase of these lipoproteins in plasma, along with insufficient LPL activity, collectively contribute to the development of coronary atherosclerosis (25).

The intricate interplay between VLDL and coronary atherosclerosis underscores the significance of VLDL metabolism in cardiovascular health, providing valuable insights into potential mechanisms underlying the relationship between metabolic disorders like T2D and the development of atherosclerosis.

### 1.5. Research landscape and scope of the study

The causal relationships between T2D, VLDL, and coronary atherosclerosis have each been independently established through 2-sample analyses (26–28). However, substantial research gaps persist in elucidating the intricate pathways that connect T2D to coronary atherosclerosis, highlighting the imperative for further investigation. Within this context, investigating the mediating role of VLDL emerges as a promising avenue of exploration.

MR emerges as a robust strategy to probe causal relationships, effectively addressing the gaps in our current understanding (29). This analytical approach, utilizing multiple IVs, holds the potential to unravel the complexity of these relationships. Through the estimation of genetic variant effects on intermediate phenotypes (such as blood glucose) and their subsequent influence on outcomes (such as coronary atherosclerosis), MR offers a pathway to uncover the underlying mechanisms linking T2D to coronary atherosclerosis.

In summary, this study's focus on elucidating the intermediate role of VLDL aims to bridge existing gaps in comprehending the intricate association between T2D and coronary atherosclerosis. Leveraging the capabilities of MR, we aspire to contribute valuable insights into the intricate mechanisms that underscore this relationship, thus advancing our understanding and presenting potential avenues for intervention and management.

## 2. Materials and methods

### 2.1. Study selection and data collection

**Figure 2:**
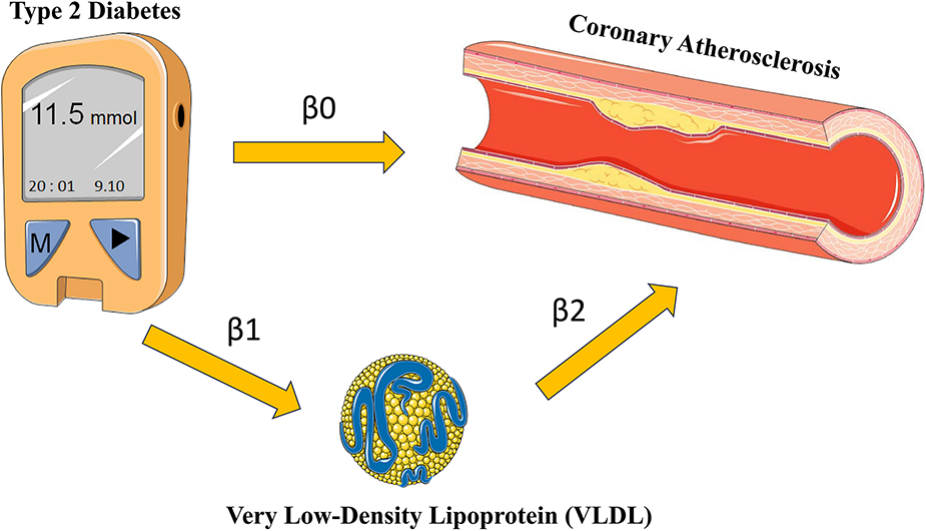
Research flow chart. Adapted from Smart Medical Art (Available at: https://smart.servier.com/).

In order to explore how T2D may contribute to the development of coronary atherosclerosis through VLDL regulation, we conducted two-sample MR analyses using data from the IEU openGWAS database (https://gwas.mrcieu.ac.uk/). The aim of these analyses was to verify the consistency of our results. We performed three MR analyses in total. The first two were conducted to investigate the causal relationship between T2D and VLDL, as well as between T2D and coronary atherosclerosis, respectively. The third analysis examined the effect of VLDL levels on coronary atherosclerosis (Figure 2). We used GWAS datasets to perform these MR analyses, and there was minimal overlap between them. Table 1 summarizes the details of the datasets used.

**Table 1:** Summarizes the details of the datasets used.

Trait	Sample size	N SNPs	Sex	Population	Year	ID
T2D	32,469/183,185	16,380,440	Males and females	Europa	2021	finn-b-E4_DM2
VLDL	115,078	12,321,875			2020	met-d-VLDL_L
Coronary atherosclerosis	23,363/195,429	16,380,466			2021	finn-b-I9_CORATHER_EXNONE

We used a significance threshold of *P *< 5e-8 to identify SNPs associated with T2D and VLDL, as this is the widely recognized standard for genome-wide association studies (GWAS) (29). To address issues related to linkage disequilibrium (LD) between the two samples, we conducted LD clumping using the TwoSampleMR package in the R language. We applied the following criteria: *R*^2 ^= 0.01 and kb = 10,000 (30). This procedure enabled the elimination of SNPs exhibiting strong LD with one another, resulting in a subset of independent SNPs for further analysis.

### 2.2. Data analysis methods

#### 2.2.1. Weak instrumental variable test

To ensure the validity of the Mendelian Randomization analysis, we implemented stringent criteria for SNP inclusion, focusing only on SNPs exhibiting strong associations with the respective exposures, namely Type 2 Diabetes or VLDL levels (9). The robustness of individual SNPs or sets of SNPs was assessed through the calculation of the F-statistic, providing a measure of the instrument strength. Additionally, we examined the proportion of variance in the exposure explained by the instrumental variable, as indicated by the *R*^2^ statistic (31). These rigorous metrics were employed to ascertain the reliability and potency of the instrumental variables utilized in our MR analysis.

The *F*-statistic, calculated as $F=\frac{N-K-1}{K}\;\times \;\frac{R^{2}}{1-\;R^{2}}$, was employed for the assessment, where “*N*” represents the sample size of the exposure, and “*K*” denotes the number of SNPs associated with both the exposure and the depth of the Genome-Wide Association Study. Furthermore, the determination of $R^{2}$ relied on the formula $2\;\;\times \;(1-\mathrm{MAF}\;)\;\;\times \;\mathrm{MAF}\;\;\times \;\;\beta^{2}$, with “MAF” representing the Minor Allele Frequency and $\beta^{2}$ signifying the effect size of the SNP on the exposure. This thorough evaluation process served to enhance the confidence in the instrumental variables used for the MR analysis.

#### 2.2.2. Causal effect estimation

In this study, we utilized multiple SNPs as instrumental variables for Mendelian Randomization (MR) analysis. To assess the association of each individual SNP, we employed the Wald statistic with the following formula (31):

$$\hat{\theta_{i}}=\frac{\hat{\beta_{i}^{y}}}{\hat{\beta_{i}^{x}}}$$

$\hat{\theta_{i}}$ represents the estimated effect size for SNP *i*
$\hat{\beta_{i}^{y}}$ denotes the effect size of the SNP on the outcome variable.
$\hat{\beta_{i}^{x}}$ represents the effect size of the SNP on the exposure variable.

To evaluate the relationship between T2D and coronary atherosclerosis, we combined Wald ratios using the inverse variance weighted (IVW) method (32). In this context, $\hat{\theta_{i}}$ represents the estimated causal effect, $\hat{\beta_{i}^{y}}$ denotes the effect size of the SNP on the outcome variable, and $\hat{\beta_{i}^{x}}$ represents the effect size of the SNP on the exposure variable. Additionally, we employed the MR-Egger regression method (33) and the weighted median estimator (WME) (34) to complement and validate the MR results.

It is important to note that the validity assumptions for the three calculation methods used for instrumental variables differ, which helps ensure the robustness of the test results. The IVW method calculates the effect estimate as the slope of a linear regression weighted on the exposure factor for the instrumental variable in the outcome, with the intercepted item constrained to be zero. If all selected SNPs are valid instrumental variables, the IVW rule can provide unbiased effect estimates. In contrast, the MR-Egger method considers the existence of pleiotropy in the instrumental variables by using an intercept term in the weighted regression. The intercept term is used to evaluate the pleiotropy between the instrumental variables, and the slope is estimated accordingly. Finally, the WME method can still estimate the causal effect even when the proportion of invalid instrumental variables is as high as 50% and the estimated precision of the instrumental variables is quite different.

To evaluate the presence of heterogeneity among the instrumental variables, we used Cochran's Q test with both the IVW and MR-Egger methods (35, 36). If there is heterogeneity among the instrumental variables, we used the IVW of the random-effects model for the analysis of the results. In contrast, if there is no heterogeneity, the IVW of the fixed-effects model is used as the main approach (36).

#### 2.2.3. Reliability evaluation

One must bear in mind that when it comes to instrumental variables, they are typically assumed to impact outcomes solely through the exposure factors being investigated. In other words, there is no direct association between these variables and the outcomes themselves. Nonetheless, this assumption becomes increasingly challenging to verify because genetic variation can exhibit pleiotropic effects—meaning that one gene may influence multiple traits or phenotypes simultaneously. Consequently, fully testing the exclusion hypothesis poses difficulties. At present, researchers widely rely on the intercept term of MR-Egger regression as a tool for detecting potential instances of pleiotropy. Essentially, if the Egger intercept (i.e., linear regression intercept) in an MR-Egger model approximates zero closely enough, it indicates a lack of evidence supporting genetic pleiotropy; thus, reinforcing the validity of the exclusionary hypothesis. Moreover, a significantly different result suggests otherwise (32, 37).

To assess the sensitivity of the results, a leave-one-out analysis was performed. This method is widely used to identify potential outliers by removing each SNP one by one and observing whether the results differ significantly before and after the removal. Specifically, if the obtained *P*-value is greater than 0.05 after excluding a particular SNP, it suggests that the SNP does not have a non-specific effect on the estimation of the causal effect (30).

## 3. Results

### 3.1. Relevance

The *F*-statistic value is all >10 in every filtering step, indicating strong instrumental variables. The threshold of *r*-squared is 0.01. The low likelihood of weak instrumental variable bias, as suggested by the *R*^2^ and *F* values, further supports the assumption of relevance in MR research.

### 3.2. Two-step Mendelian randomization results

**Figure 3:**
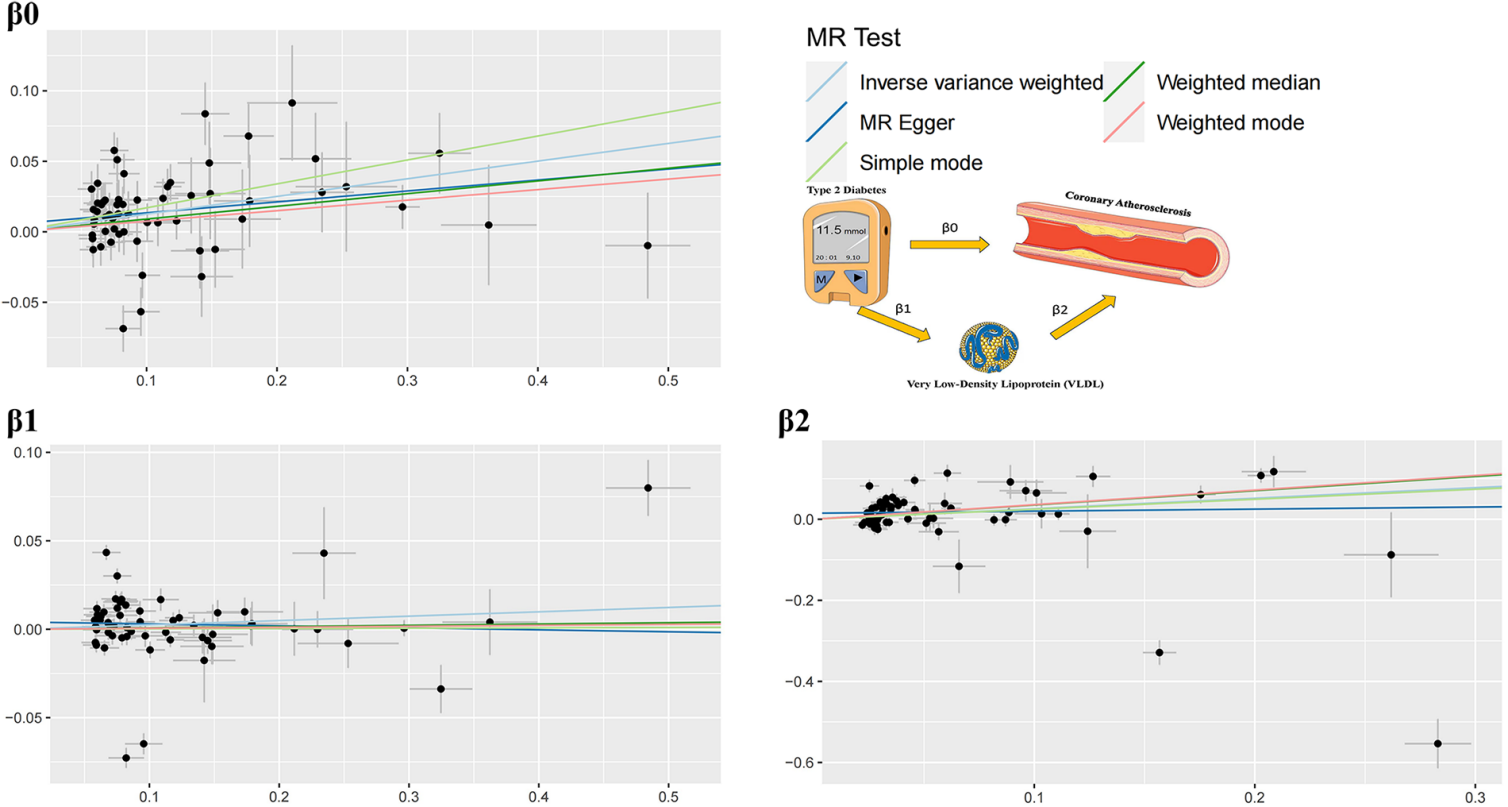
MR analysis.

**Figure 4:**
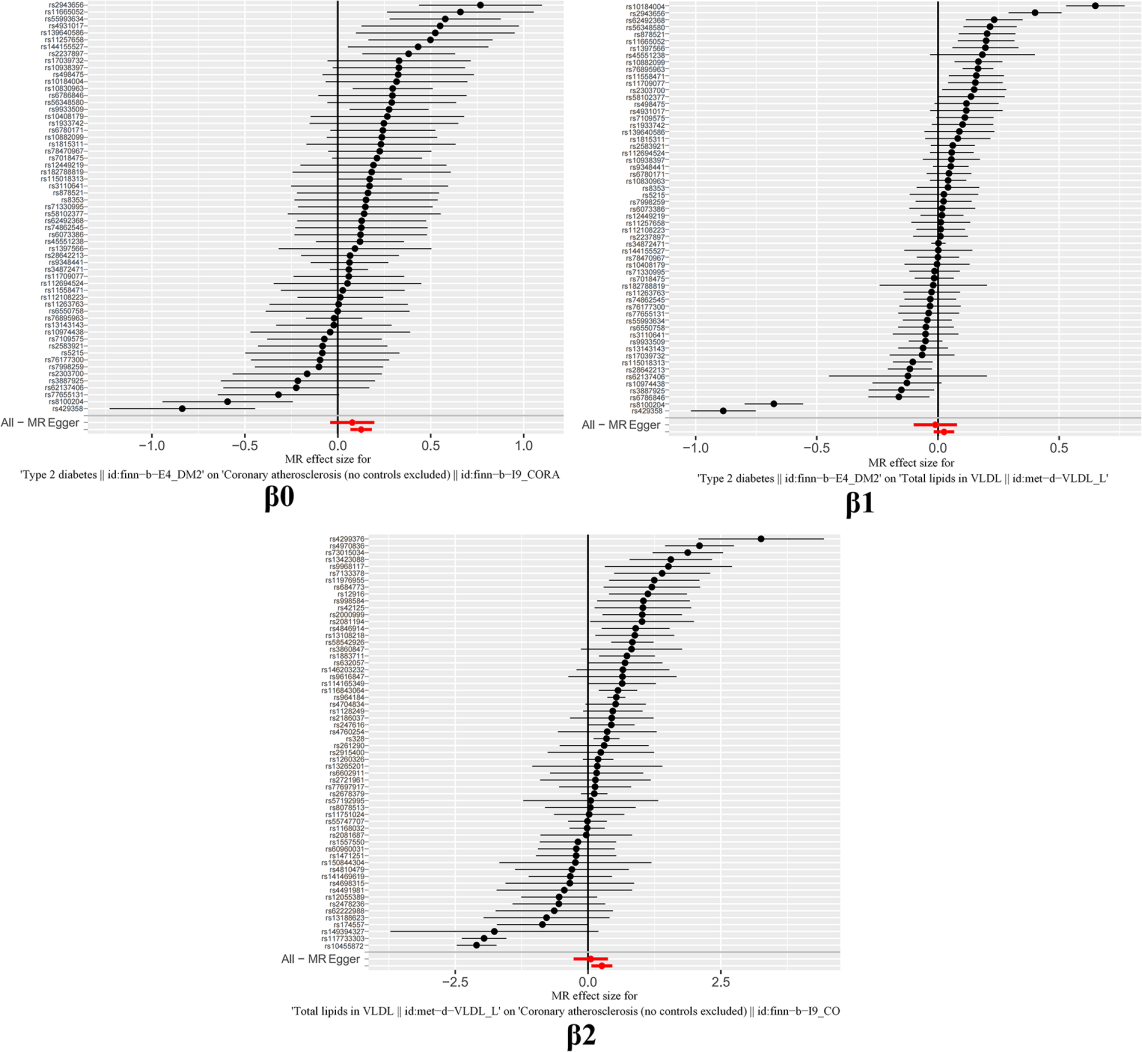
Forest plots of MR analysis.

**Figure 5:**
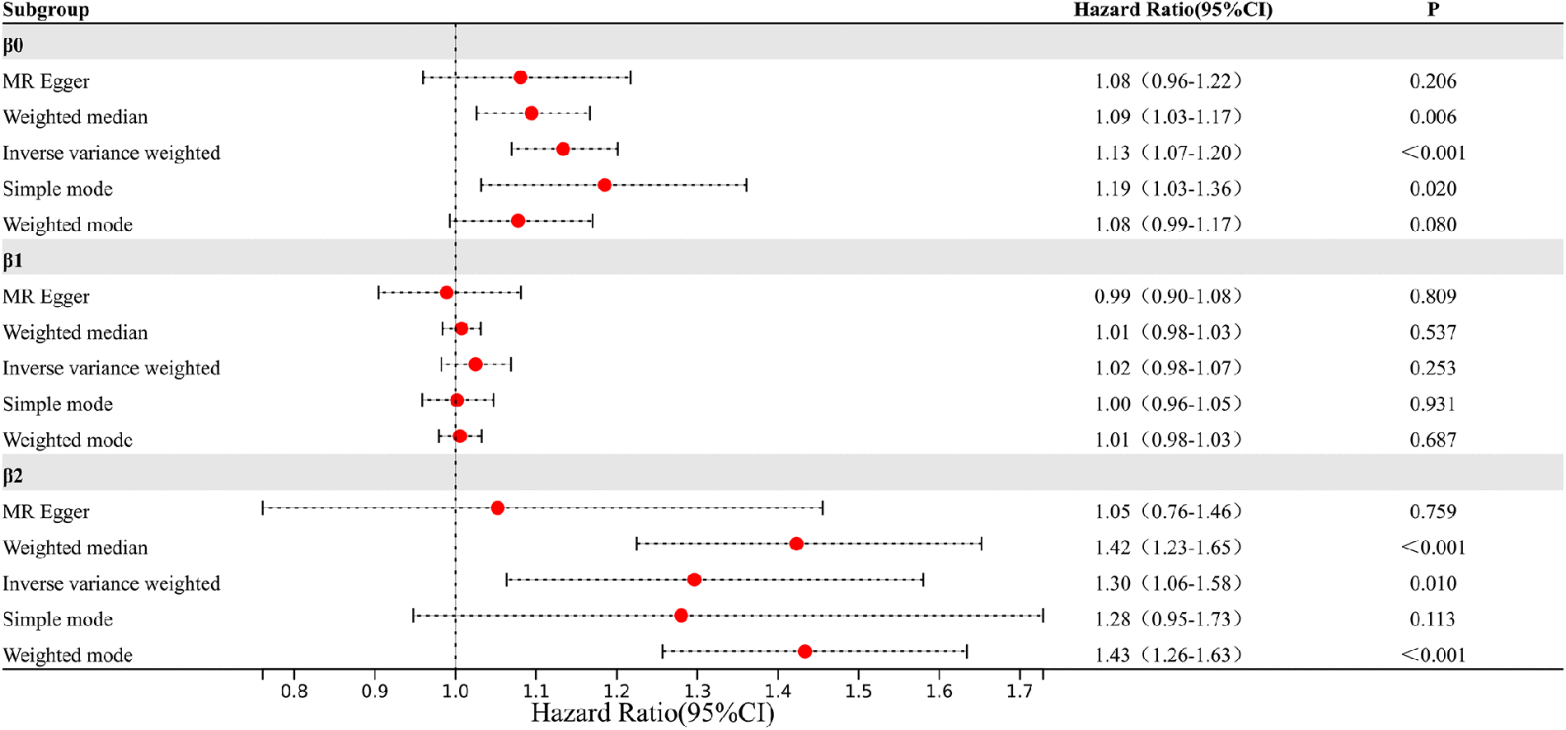
The result of 5 methods and 2 tests.

The study findings, depicted in Figures 3, 4 and summarized in Figure 5, reveal the established causal links between the exposures and outcomes evaluated through MR-Egger regression, weighted median, and random effects inverse variance weighting methods. In addition, assessments for heterogeneity and horizontal pleiotropy were executed, with the respective outcomes presented in Figure 5. While the heterogeneity test results might not align optimally, possibly attributable to the intricate pathogenesis of T2D, they do not undermine the overarching conclusion.

The analysis reveals significant findings across multiple methods and tests investigating the relationships between various factors. Notably, for *β*^0^, which pertains to the causal impact of T2D on the development of coronary atherosclerosis, the inverse variance weighted method indicates a substantial association with an odds ratio (OR) of 1.13 [95% confidence interval (CI): 1.07–1.20], highlighting the elevated risk of coronary atherosclerosis due to T2D. Likewise, the relationship between Type 2 diabetes and VLDL levels, denoted as *β*^1^, exhibited modest associations across the various methods employed. These results imply that while a direct influence of T2D on VLDL levels is observed, it does not reach statistical significance in most analyses. This suggests a nuanced connection that might contribute to the intricate interplay between T2D and VLDL in the context of cardiovascular risk factors. Regarding *β*^2^, which signifies the relationship between VLDL levels and the occurrence of coronary atherosclerosis, the weighted median method demonstrated a significant odds ratio of 1.42, indicating that higher VLDL levels significantly increase the likelihood of developing coronary atherosclerosis. The inverse variance weighted method also presented a meaningful association with an odds ratio of 1.30, further underlining the role of VLDL in the development of coronary atherosclerosis.

The outcomes of horizontal pleiotropy assessment (Figure 4) depicted in these three figures serve as a means of mitigating horizontal pleiotropy, a factor that must be accounted for in Mendelian randomization analyses. Horizontal pleiotropy refers to effects that must be eliminated in Mendelian randomization, as each individual SNP locus can potentially exhibit horizontal pleiotropy. The overall pleiotropy fit observed in the images converges closely to zero, thus statistically implying the absence of horizontal pleiotropy.

It is important to note that the conclusions drawn from MR analysis are based on several assumptions, including the validity of instrumental variables, the absence of horizontal pleiotropy, and the absence of unmeasured confounding factors. While efforts were made to ensure the validity of instrumental variables, it is still possible that some SNPs may have pleiotropic effects or be subject to weak instrument bias. Therefore, the results should be interpreted with caution, and further studies are needed to confirm the causal relationship between T2D, VLDL, and coronary atherosclerosis.

### 3.3. Reliability evaluation

**Figure 6:**
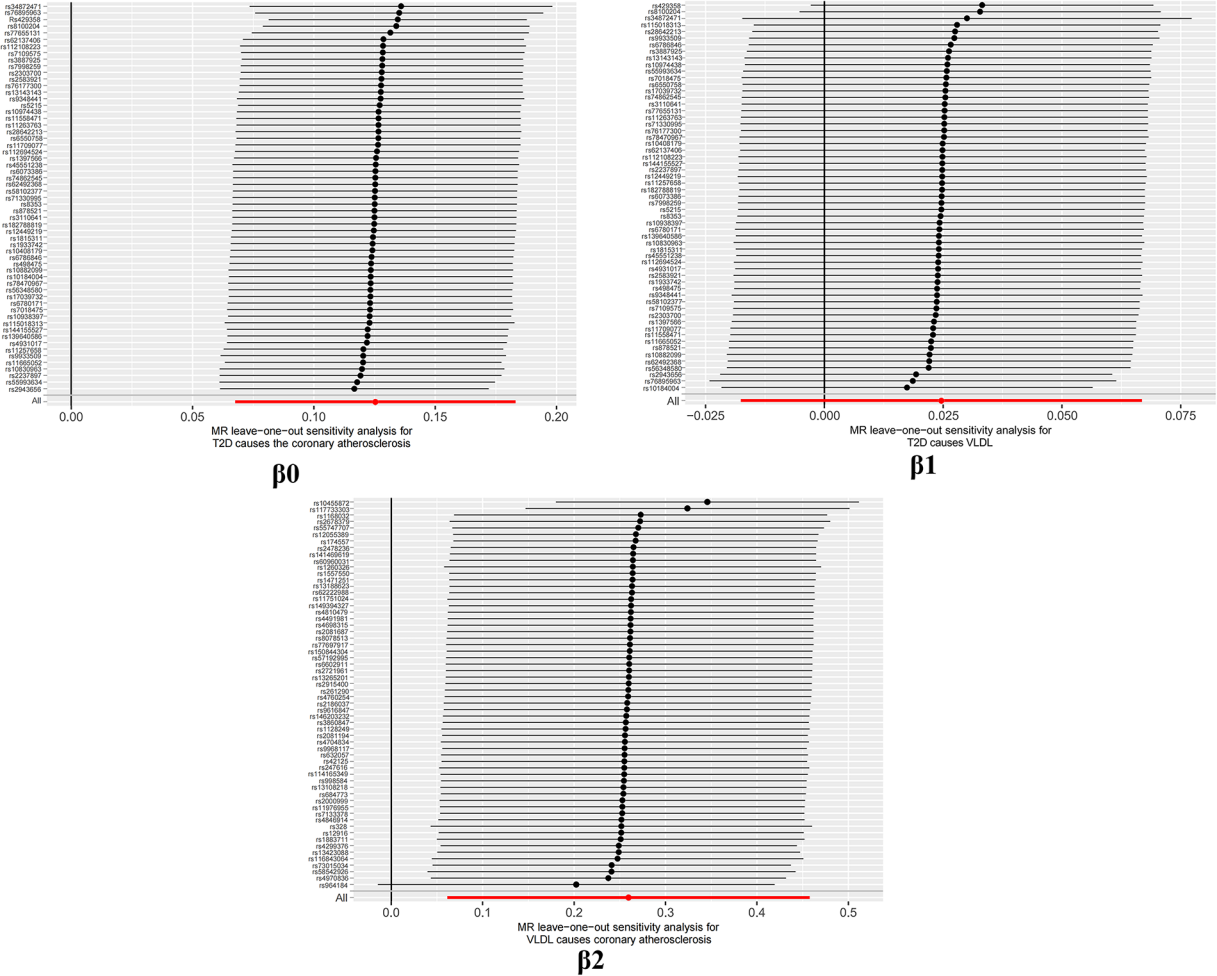
“leave one out” sensitivity analysis.

It is worth highlighting that the “Leave-one-out” sensitivity analysis should be conducted across all instrumental variables employed in the analysis, extending beyond the six groups of data mentioned earlier. Despite the positive outcomes currently depicted in Figure 6, this analysis should be iteratively repeated by excluding each individual instrumental variable to assess its impact on the overall results. This meticulous approach offers additional confirmation that the favorable results are not reliant on a single SNP or a limited subset of SNPs.

## 4. Conclusion

Cardiovascular disease (CVD) is a global health concern with significant morbidity and mortality (38). Within its range, coronary heart disease (CHD) significantly impacts individuals with T2D, being a key contributor to morbidity and mortality (39). T2D is an established risk factor for CHD, with hyperglycemia directly triggering coronary atherosclerosis (40). Elevated blood glucose also contributes to VLDL buildup (41). Particularly important is the link between dyslipidemia—characterized by VLDL and high TG levels—and CHD (42). This investigation aims to uncover VLDL's role, address knowledge gaps, and enhance understanding of the complex T2D and coronary atherosclerosis relationship via Mendelian randomization.

This study utilized large-scale GWAS meta-analysis data and employed a two-sample Mendelian randomization approach to investigate the causal relationships between T2D, VLDL, and coronary atherosclerosis. T2D increases the risk of developing coronary atherosclerosis, leading to a 13.35% increase in disease occurrence compared to individuals without T2D. Additionally, in the context of patients with T2D, VLDL concentration increases by 2.49%. For every one standard deviation increase in VLDL, the probability of developing heart disease increases by 29.6%. These findings suggest that VLDL may serve as a mediator in the link between T2D and coronary atherosclerosis.

According to reports, coronary atherosclerosis is a significant global health concern, particularly among individuals with T2D due to their elevated risk of CHD (43). VLDL, intricately linked with CHD risk, plays a pivotal role in this context. T2D is known to elevate CHD risk through mechanisms such as chronic inflammation, insulin resistance, and oxidative stress, all of which contribute to the development of atherosclerosis—an underlying factor in CHD progression (44, 45). Moreover, VLDL, a central risk factor for CHD, assumes a crucial role (46). Elevated VLDL levels, characteristic of dyslipidaemia, independently elevate CHD risk by promoting atherosclerosis development (47). This study focused on understanding the individual influences of T2D and VLDL levels on coronary atherosclerosis risk and explored the potential mediating role of VLDL (47). The findings not only delineated the separate contributions of T2D and VLDL levels to coronary atherosclerosis risk but also proposed that VLDL might operate as a mediating pathway. These findings accentuate the significance of managing VLDL levels to mitigate the onset of coronary atherosclerosis among T2D individuals, underlining the need for early intervention to manage CHD risks.

This study possesses several strengths. Firstly, it leveraged large-scale GWAS databases and incorporated hundreds of SNPs in each two-sample Mendelian randomization analysis, minimizing the potential for random outcomes and enhancing the proportion of variance explained by the SNPs. Additionally, the study's robustness is underscored by conducting GWAS for all three variables using European databases with a low overlap probability, effectively addressing the concern of population bias. Furthermore, unlike similar studies focused solely on specific populations, this research significantly broadened its scope by encompassing a diverse European database, contributing to the generalizability of its findings.

While employing the two-sample Mendelian randomization method, this study demonstrates notable strengths as well as certain limitations. Firstly, despite the utilization of GWAS data spanning European databases, the extent of overlap remains low, potentially impacting the external applicability of the findings. Additionally, the assumption of method validity encompasses the effectiveness of instrumental variables; however, the presence of weak instruments might introduce inaccuracies in estimations. On another note, the study might have some shortcomings in controlling for confounding factors, such as lifestyle, genetics, and other potential covariates. This could potentially affect the internal validity of the results, making it challenging to completely exclude the influence of other factors. Furthermore, constrained by sample size and effect magnitude, the study's statistical power could be limited, leading to a potential weakening of result stability. Therefore, careful interpretation of the generalizability of the findings is warranted. These limitations underscore the need for cautious interpretation and highlight avenues for future research.

In conclusion, this study employed three two-sample Mendelian randomization analyses to investigate the relationships between T2D, VLDL, and coronary atherosclerosis. The results suggest that VLDL may potentially serve as a mediator in the pathway through which T2D leads to coronary atherosclerosis. This innovative approach bridges the gap between experimental and genomic methodologies, providing robust evidence for the causal link between these conditions. By incorporating VLDL levels as a potential mediating factor, it unveils a previously unexplored facet of their interplay, shedding light on the intricate mechanisms underlying this complex association.

The findings of this research have significant implications for clinical practice and public health policy formulation. Confirming the mediating role of VLDL in the T2D-coronary atherosclerosis association underscores the importance of reducing VLDL levels, potentially aiding in coronary atherosclerosis risk reduction. Due to the intricate interplay between T2D and coronary atherosclerosis, these results can guide the development of targeted intervention strategies, facilitating early identification and treatment of abnormal VLDL levels in T2D patients, thereby mitigating cardiovascular risks. Additionally, these discoveries offer a roadmap for future investigations, motivating further exploration into the mechanisms underlying T2D, VLDL, and coronary atherosclerosis, consequently providing more precise and effective approaches for cardiovascular disease prevention and management. This study not only enhances our understanding of the mechanisms underlying relevant diseases but also provides valuable insights for the realms of clinical practice and public health.

## Data Availability

The datasets presented in this study can be found in online repositories. The names of the repository/repositories and accession number(s) can be found in the article/Supplementary Material.
